# A Transcriptomic Approach to the Metabolism of Tetrapyrrolic Photosensitizers in a Marine Annelid

**DOI:** 10.3390/molecules26133924

**Published:** 2021-06-27

**Authors:** Maria Leonor Santos, Mariaelena D’Ambrosio, Ana P. Rodrigo, A. Jorge Parola, Pedro M. Costa

**Affiliations:** 1 UCIBIO–Applied Molecular Biosciences Unit, Department of Life Sciences, NOVA School of Science and Technology, FCT-NOVA, NOVA University of Lisbon, 2829-516 Caparica, Portugal; mlve.santos@campus.fct.unl.pt (M.L.S.); m.dambrosio@fct.unl.pt (M.D.); a.rodrigo@campus.fct.unl.pt (A.P.R.); 2 LAQV–Associate Laboratory for Green Chemistry, Department of Chemistry, NOVA School of Science and Technology, FCT-NOVA, NOVA University of Lisbon, 2829-516 Caparica, Portugal; ajp@fct.unl.pt

**Keywords:** porphyrin metabolism, photodynamic, heme, bile pigments, Annelida, bioinformatics

## Abstract

The past decade has seen growing interest in marine natural pigments for biotechnological applications. One of the most abundant classes of biological pigments is the tetrapyrroles, which are prized targets due their photodynamic properties; porphyrins are the best known examples of this group. Many animal porphyrinoids and other tetrapyrroles are produced through heme metabolic pathways, the best known of which are the bile pigments biliverdin and bilirubin. *Eulalia* is a marine Polychaeta characterized by its bright green coloration resulting from a remarkably wide range of greenish and yellowish tetrapyrroles, some of which have promising photodynamic properties. The present study combined metabolomics based on HPLC-DAD with RNA-seq transcriptomics to investigate the molecular pathways of porphyrinoid metabolism by comparing the worm’s proboscis and epidermis, which display distinct pigmentation patterns. The results showed that pigments are endogenous and seemingly heme-derived. The worm possesses homologs in both organs for genes encoding enzymes involved in heme metabolism such as ALAD, FECH, UROS, and PPOX. However, the findings also indicate that variants of the canonical enzymes of the heme biosynthesis pathway can be species- and organ-specific. These differences between molecular networks contribute to explain not only the differential pigmentation patterns between organs, but also the worm’s variety of novel endogenous tetrapyrrolic compounds.

## 1. Introduction

Natural pigments are chemically diverse derivatives of metabolic products of biosynthetic or catabolic pathways. In the past decade, marine natural pigments have gained attention in biomedicine due to their antioxidant, antimicrobial, and photoprotective properties [1,2]. Despite the acknowledged importance of shallow-water marine invertebrates, known for their bright coloration, as source of novel pigments, explorations of the biotechnological potential of these pigments are lagging [1]. Among these organisms, the Polychaeta are a promising target for bioprospecting of novel marine bioproducts, informed by their diversity, abundance, and promising findings to date (see Rodrigo & Costa [3] for a review).

Tetrapyrroles are one of the most abundant naturally occurring pigment groups [4]. This class of organic compounds is characterized by four pyrrolic rings which are usually arranged in a cyclic configuration. Their most well-known representatives are chlorins and porphyrins and their derivatives, which include chlorophyll and heme, respectively [5]. These compounds have a distinctive absorption spectrum, characterized by strong absorption between approximately 380 and 500 nm (the Soret band), followed by four weaker absorption maxima between 500 and 750 nm, called the Q bands [6]. Compounds with tetrapyrrolic structures, such as chlorins and porphyrins, are often photoactive and are therefore suitable photosensitizers, i.e., compounds that absorb light and generate reactive oxygen species (ROS) in the presence of oxygen [7,8]. These unique characteristics make these pigments of high interest in photodynamic therapy (PDT), a treatment that uses photosensitizers for targeted cytotoxicity against malignancies or infections, especially of the skin, with particular emphasis on melanomas [9]. Interestingly, one of the first works on tetrapyrrolic pigments focused on the Polychaeta (Echiura) *Bonellia viridis*. Its unique green pigment (bonellin) is a chlorin (unrelated to chlorophyll) for which photodynamic activity has been reported [10].

Bile pigments are endogenous secondary metabolites from the (enzyme-mediated) catabolism of heme, the best known of which are biliverdin and bilirubin [11,12]. Heme is a ubiquitous prosthetic group composed of iron and protoporphyrin IX; it is an essential component of biological structures such as hemoglobin, peroxidases, and cytochromes of the P450 family [13]. In turn, canonical heme biosynthesis is described as an eight-step, well-conserved, enzymatic cascade that occurs in mitochondria and in cytosol (see Heinemann et al. [13] for more details). The stepwise sequence of enzymes is as follows: δ-aminolevulinic acid synthase (ALAS), δ-aminolevulinic acid dehydratase (ALAD), hydroxymethylbilane synthase (HMBS), uroporphyrinogen synthase (UROS), uroporphyrinogen decarboxylase (UROD), coproporphyrinogen oxidase (CPOX), protoporphyrinogen oxidase (PPOX), and ferrochelatase (FECH). The porphyrinoid intermediates of the heme biosynthetic pathway are potentially toxic (including heme itself, as it can be a source of ROS), which implies tight regulation to avoid the accumulation of hazardous byproducts [14,15]. Conversely, the breakdown of heme can occur through several mechanisms. One of the best known pathways is the catabolism of heme in the reticuloendothelial system of mammals, which involves the heme oxygenase (HMOX) enzyme complex [16]. Heme is converted by HMOX to biliverdin, a green pyrrolic product that is rapidly metabolized by biliverdin reductase (BLVRA) to bilirubin, a yellow pigment found in the bile of vertebrates that is eliminated at the duodenum [16,17]. In Polychaeta, endogenous biliverdin has been reported in *Hediste* (*Nereis*) *diversicolor* [18]. However, the metabolic pathways of heme-related pigments in Polychaeta are still mostly unknown.

The members of the genus *Eulalia* (*E. viridis*/*clavigera*) are marine Polychaeta known for their bright green coloration and mainly found on Atlantic rocky shores, usually seeking protection underneath mussel beds [19]. These worms bear specialized pigment cells in the proboscis and epidermis which produce greenish pigment granules that can also be found in the intestine, suggesting that the pigments responsible for the worm’s green coloration are, at least in part, metabolized and eliminated in the digestive epithelia [20,21]. Martins et al. [22] identified the worm’s endogenous pigments as greenish or yellowish tetrapyrroles, the distribution and abundance of which differ between organs. These authors reported that while the proboscis holds mostly yellow pigments, the epidermis has both, with the majority being green pigments, whereas intestine and oocytes only yield green pigments. In contrast to *Hediste diversicolor*, which is known for its widely variable pigmentation pattern [18], tetrapyrroles in *Eulalia* constitute its primary coloration. Most importantly, D’Ambrosio et al. [23] revealed photodynamic properties in *Eulalia*’s tetrapyrrolic pigments, which, together with the wide range of pigments, makes the worm (and likely other Polychaeta) a promising target for bioprospecting for novel photosensitizers. Photosensitizer pigment synthesis in vitro is a laborious and costly endeavor. Knowing whether and how these pigments can be synthesized in vitro, whether through the use of cost-effective biological models (such as bacteria, yeast, or animal cell cultures) or enzymatic and nonenzymatic catalysts able to mimic the biosynthetic process, would offer a major advantage for biopharmaceutical applications with respect to cost and availability. Such knowledge requires the unveiling not only of the chemistry of the pigments per se, but also of the metabolic pathways leading to their formation and biotransformation, which inherently involves the identification of key enzymes and their variants. While previous studies have suggested that the pigments of *Eulalia* are products of heme breakdown [20,21,22,23], the current work aimed to understand the mechanisms of porphyrinoid and related pigment metabolism in *Eulalia* by identifying the main enzymes and gene networks involved in heme synthesis and catabolism; to compare between the main pigment-bearing organs, the proboscis and epidermis; and to make inferences about the potential roles of the pigments as candidates for biotechnological applications.

## 2. Results

### 2.1. Characterization of Tetrapyrrolic Compounds

**Figure 1:**
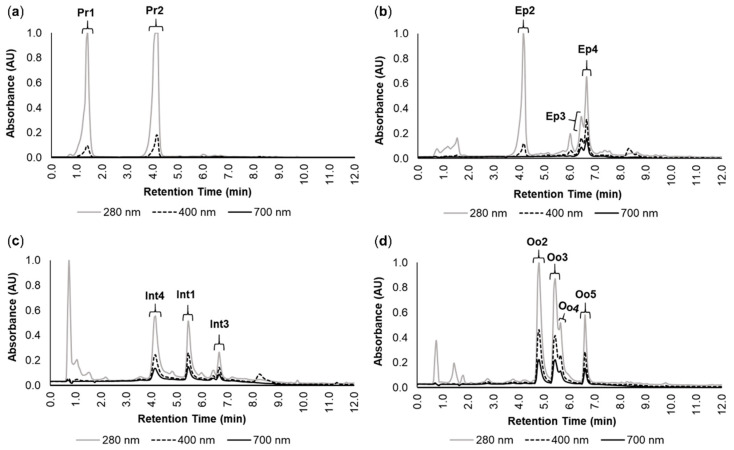
Main pigments in the studied organs of *Eulalia*. The data were retrieved by high-performance liquid chromatography with a diode array detector (HPLC-DAD). Absorbance (AU) and retention time (min) are illustrated for each chromatogram for the extracts from (**a**) proboscis, (**b**) epidermis, (**c**) intestine, and (**d**) oocytes. Each pigment was labeled according to Martins et al. [22]. The selected wavelengths were 280, 400, and 700 nm to fit absorbance within the range of UV, violet (to detect yellow pigments), and red (to detect green pigments), respectively.

The chromatograms of the crude pigment extracts from the proboscis, epidermis, intestine, and oocytes revealed multiple absorption maxima, with strong absorption in the UV zone (280 nm) as well as in the violet (400 nm) and red (700 nm) regions of the visible light spectrum (Figure 1). According to Martins et al. [22], twelve main individual pigments were identified and labeled. The pigments from the proboscis (Figure 1a) bore a yellowish tone and were termed Pr1 and Pr2, with both having absorption maxima at 280 nm and high absorption at 400 nm (Figure 1a). The retention times of Pr1 and Pr2 were 1.04 to 1.63 min and 3.69 to 4.34 min, respectively. The chromatogram from the epidermis (Figure 1b) also revealed a yellowish pigment, termed Ep2, with a retention time (3.85 to 4.25 min) and absorbance similar to those of Pr2 (Figure 1b). Two additional green pigments named Ep3 and Ep4 were identified in the epidermis, with overlapping retention times (from 6.36 to 6.76 min). Both these pigments yielded absorption maxima at 280 and high absorption at 400 and 700 nm (Figure 1b). The intestine and oocytes revealed several greenish pigments with absorption maxima at 280 nm, and high absorption at 400 and 700 nm (Figure 1c,d). Pigments from the intestine (Int1, Int3, and Int4) had retention times between 4.01 and 6.72 min (Figure 1c). In turn, the oocytes displayed four major pigments (Oo2, Oo3, Oo4, and Oo5) with retention times between 4.70 and 6.70 min (Figure 1d).

**Figure 2:**
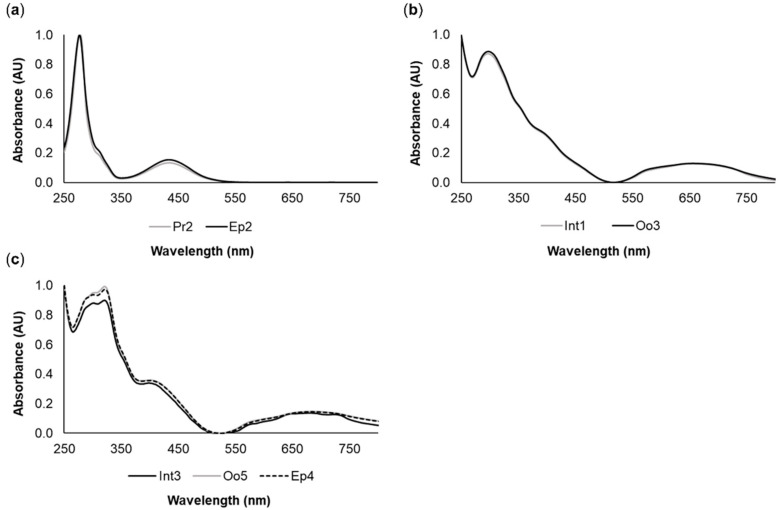
Common pigments between the different organs of *Eulalia*. The spectra were produced by high-performance liquid chromatography with a diode array detector (HPLC-DAD). (**a**) Yellow pigments Pr2 (proboscis) and Ep2 (epidermis); (**b**) Green pigments Int1 (intestine) and Oo3 (oocytes); (**c**) Green pigments Int3 (intestine), Oo5 (oocytes), and Ep4 (epidermis).

The yellow pigments Pr2 (proboscis) and Ep2 (epidermis), in addition to having similar retention times, also displayed identical absorption spectra (Figure 2a), which indicated that they are chemically related. Similarly, the green pigments Int1 (intestine) and Oo3 (oocytes) also yielded similar retention times and matching absorption spectra (Figure 2b). A similar result was found for the green pigments Int3, Oo5, and Ep4 from the intestine, oocytes, and epidermis, respectively (Figure 2c). Pigment characterization is summarized in Table 1.

**Figure 3:**
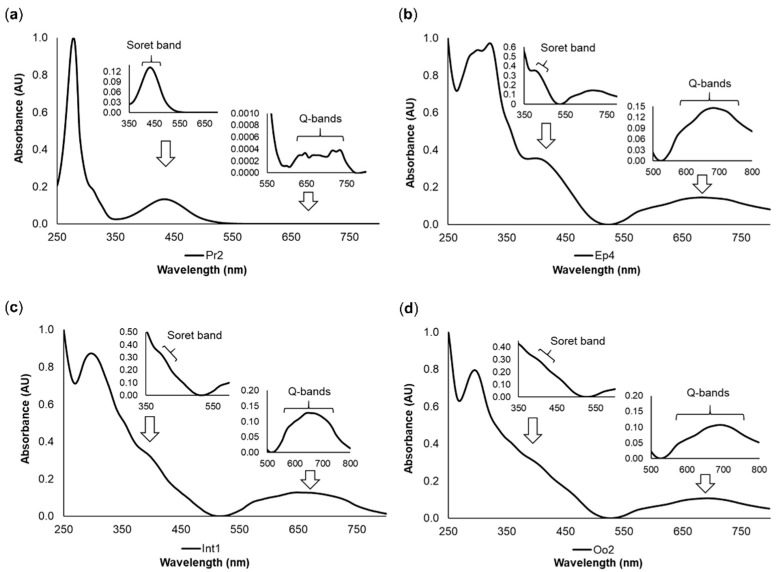
Representative pigment absorption spectra of *Eulalia* pigments. The data were retrieved using high-performance liquid chromatography with a diode array detector (HPLC-DAD). Each graph displays the Soret band (380–500 nm) and Q bands (500–750 nm) with different degrees of resolution. (**a**) Pr2, a yellow pigment from the proboscis; (**b**) Ep4, a green pigment from the epidermis; (**c**) Int1, a green pigment from the intestine; and (**d**) Oo2, a green pigment from the oocytes. The retention times were 4.05 min for Pr2, 6.63 min for Ep4, 5.47 min for Int1, and 4.89 min for Oo2.

The tetrapyrrolic nature of pigments was verified by the presence of two sets of bands in the visible region, one centered at 380–500 nm and the other at 500–750 nm, as shown in Figure 3. These features presented high resolution in the yellow pigment Pr2 (Figure 3a), while for the green pigments Ep4, Int1, and Oo2, less resolved, broader bands were observed (Figure 3b,c,d respectively). The absorption spectra of the remaining pigments showed the same differences between yellowish and greenish pigments (data not shown). The more structured bands resembled the Soret and Q bands of porphyrinoid compounds, suggesting the presence of cyclic tetrapyrroles either as main components or as traces of precursor compounds.

### 2.2. Gene Expression Profiling between the Proboscis and Epidermis

**Figure 4:**
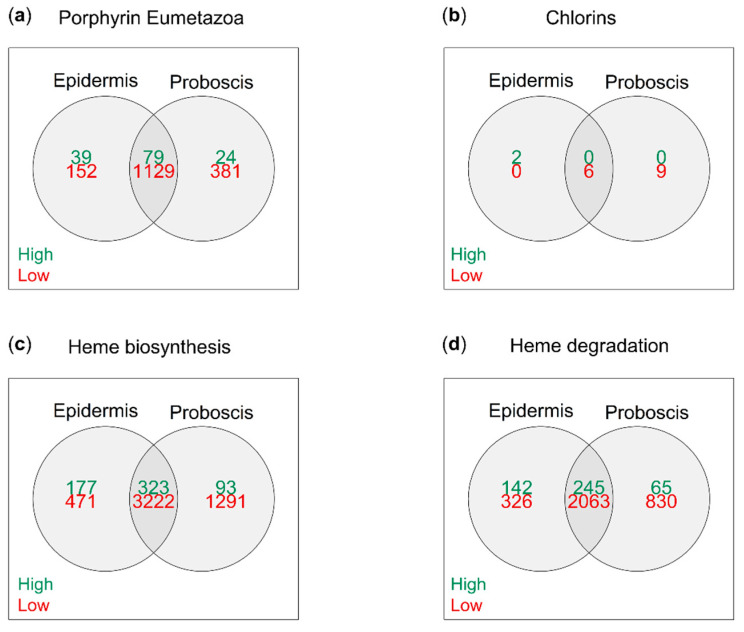
Relative expression of total tetrapyrrole/porphyrinoid-related ORFs in *Eulalia* obtained via RNA-seq and de novo transcriptome assembly. Each Venn diagram represents a customized subset of the Uniprot database built from specific search terms. (**a**) “Porphyrin Eumetazoa”, (**b**) “chlorins”, (**c**) “heme biosynthesis”, and (**d**) “heme degradation”. Relative expression was arbitrarily identified as “high” and “low” when logTPM > 2 and logTPM < −2, respectively.

De novo transcriptome assembly using Trinity yielded a total of 55,508 individual open reading frames (ORFs) in *Eulalia*. The translated transcripts were annotated based on homology matching against four subsets of the Uniprot protein database associated with the search terms “chlorins”, “porphyrin Eumetazoa”, “heme biosynthesis”, and “heme degradation”. The subsets that provided most matches were “heme biosynthesis” and “heme degradation”, with 7198 and 4651 matched proteins (maximum *e*-value of 10^−5^), respectively, followed by “porphyrin Eumetazoa” with 2294 and, lastly, “chlorins” with only 32. On the other hand, the preceding subsets, heme biosynthesis, heme degradation, and porphyrin Eumetazoa yielded 593, 452, and 142 proteins, respectively, which matched with relatively abundant (logTPM > 2) mRNAs (Figure 4).

**Figure 5:**
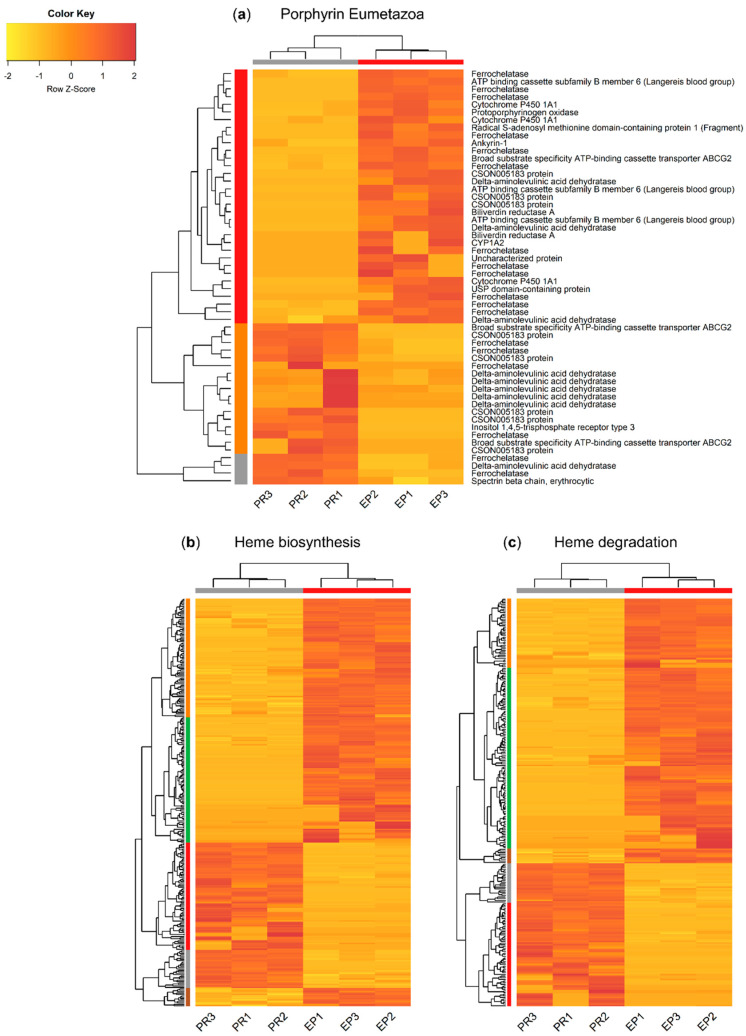
Heat maps illustrating the overall patterns of differentially transcribed mRNAs potentially pertaining to genes associated with the metabolism of tetrapyrroles and related compounds between the proboscis and epidermis of *Eulalia*. Separated by annotation subset retrieved from Uniprot. Three independent replicates (corresponding to three different individuals) per organ are represented (PR1-3 and EP1-3 for proboscis and epidermis, respectively). (**a**) “Porphyrin Eumetazoa”, (**b**) “heme biosynthesis”, and (**c**) “heme degradation”. Horizontal and vertical dendrograms plus sidebars represent clustering of biological replicates and genes, respectively. The criteria for the selection of DEGs were significant homology-matching (*e*-value < 10^−5^), FDR-adjusted *p* ≤ 0.05, and |logFC| > 2. Expression levels were row-normalized and are given as transcripts per million (TPM) and plotted as a color gradient from yellow (lowest) to red (highest). Cluster analysis was based on Euclidean distances as metric and dendrograms were built using complete linkage.

The three main subsets, “porphyrin Eumetazoa”, “heme biosynthesis”, and “heme degradation”, yielded distinct patterns of differentially expressed genes (DEGs) between the proboscis and epidermis (FDR-adjusted *p*-value < 0.05 and |logFC| > 2), as illustrated by the vertical dendrograms in Figure 5. All subsets yielded more upregulated transcripts in the epidermis than in the proboscis. Cluster analysis revealed the expected grouping of the replicate samples from the proboscis and the epidermis (horizontal dendrograms), despite intraspecific variation. The “porphyrin Eumetazoa” subset yielded 54 DEGs (Figure 5a). More δ-aminolevulinic acid dehydratase (ALAD) transcriptional variants were found in the proboscis, whereas the inverse pattern was noted for ferrochelatase (FECH). Still, the subsets with the highest number of DEGs were “heme biosynthesis” and “heme degradation” with 289 and 248 transcripts, respectively (Figure 5b,c).

### 2.3. Heme Metabolic Pathways in the Proboscis and Epidermis

**Figure 6:**
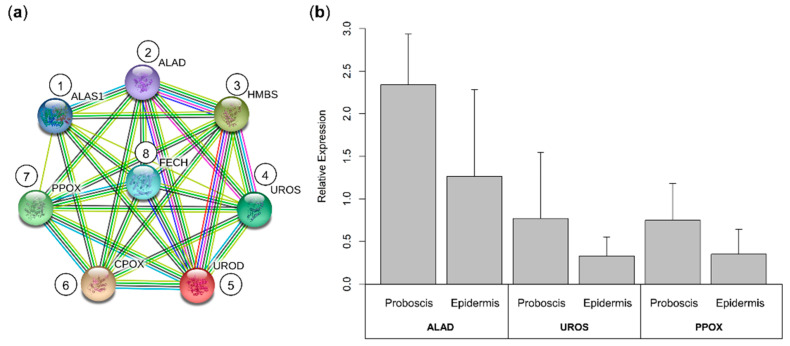
Interaction network of the enzymes involved in heme biosynthesis in the marine annelid *Eulalia* and validation of RNA-seq by RT-qPCR of a gene subset. (**a**) The eight enzymes involved in the heme biosynthesis pathway were identified in the translated transcriptome. (1) ALAS1, δ-aminolevulinic acid synthase 1; (2) ALAD, δ-aminolevulinic acid dehydratase; (3) HMBS, hydroxymethylbilane synthase; (4) UROS, uroporphyrinogen synthase; (5) UROD, uroporphyrinogen decarboxylase; (6) CPOX, coproporphyrinogen oxidase; (7) PPOX, protoporphyrinogen oxidase; and (8) FECH, ferrochelatase. Protein–protein interactions were retrieved from the STRING database with information from the “heme biosynthesis” subset. (**b**) Validation of RNA-seq data was done for mRNAs coding for ALAD, UROS, and PPOX, using RT-qPCR. Relative expression was determined via the 2^−∆∆Ct^ method following the use of primers designed to amplify the best homology-matched sequences. The results are presented as mean + standard deviation. Despite the trend for overexpression of selected genes in the proboscis, no statistical differences were found between the organs, in accordance with the RNA-seq results.

The eight fundamental enzymes involved in the canonical heme biosynthesis pathway were identified in the translated transcriptome of *Eulalia*, namely ALAS1, ALAD, HMBS, UROS, UROD, CPOX, PPOX, and FECH (Figure 6).

**Figure 7:**
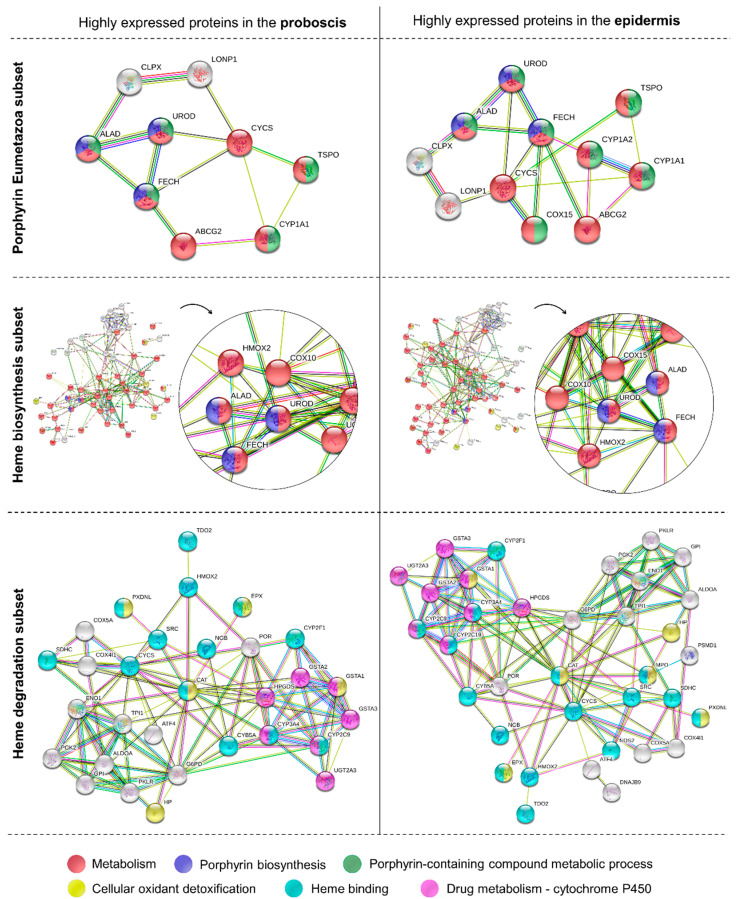
Protein–protein interactions and respective pathways or functions retrieved for the most abundant transcripts in the proboscis and epidermis. Schematic representation of the potentially highly expressed genes coding for proteins in the “porphyrin Eumetazoa”, “heme biosynthesis”, and “heme degradation” subsets. The criterion to select abundant transcripts was logTPM > 2. The various enriched pathways or functions of the proteins (based on GO terms) are represented by a different color, all with FDR-adjusted *p* < 0.05. Functional annotation and protein–protein interactions were retrieved from the STRING database.

The analysis of metabolomic pathways precluded the transcripts coding for proteins in the proboscis and epidermis yielding relatively high expression values (logTPM > 2). Overall, the enriched pathways or functions of the proteins were the same between the proboscis and epidermis, illustrated by ALAD, UROD, and FECH (Figure 7). The main enzymes in the “porphyrin Eumetazoa” and “heme biosynthesis” subsets were found to be parts of general metabolism and porphyrin biosynthesis. On the other hand, “heme degradation” proteins were mainly parts of drug metabolism and heme-binding pathways identified by GO terms. The “heme biosynthesis” and “heme degradation” subsets also displayed proteins with roles in cellular oxidant detoxification. Protein networks revealed a higher number of highly expressed proteins in the epidermis compared to the proboscis. A highly expressed protein in both organs was cytochrome P450 1A1 (CYP1A1). In contrast, cytochrome P450 1A2 (CYP1A2) and cytochrome c oxidase assembly protein COX15 homolog (COX15), were only found to be highly expressed in the epidermis. Both organs also revealed ATP-dependent Clp protease ATP-binding subunit clpX-like, mitochondrial (CLPX); broad substrate specificity ATP-binding cassette transporter (ABCG2); translocator protein (TSPO), and cytochrome c (CYCS) to be highly expressed. Similarly, heme oxygenase 2 (HMOX2) and protoheme IX farnesyltransferase (COX10) were also found to be highly expressed in both organs. The “heme degradation” subset yielded highly expressed genes in both organs, namely those coding for HMOX2, glutathione S-transferase A1 (GSTA1); glutathione S-transferase A2 (GSTA2); glutathione S-transferase A3 (GSTA3); cytochrome P450 2C9 (CYP2C9); cytochrome P450 3A4 (CYP3A4); hematopoietic prostaglandin D synthase (HPGDS); and UDP-glucuronosyltransferase 2A3 (UGT2A3).

### 2.4. Heme Biosynthesis Homologs in Metazoa

**Figure 8:**
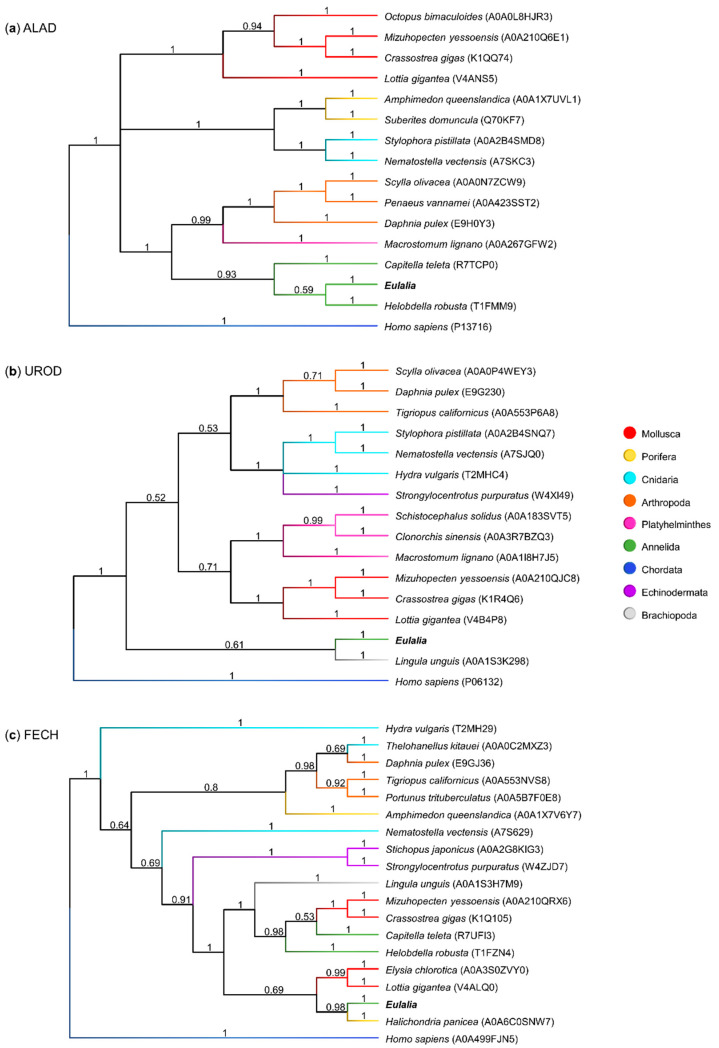
Phylogenetic trees comparing the sequences of three main heme biosynthesis proteins. Each dendrogram was created using the maximum likelihood method and an LG gamma distribution model (consensus tree obtained from 1,000,000 iterations). The sequences used were from *Eulalia* predicted transcriptome and Metazoa sequences from Uniprot for (**a**) ALAD, δ-aminolevulinic acid dehydratase; (**b**) UROD, uroporphyrinogen decarboxylase; and (**c**) FECH, ferrochelatase.

Three core heme biosynthesis proteins, ALAD, UROD, and FECH, were found to be highly expressed in both proboscis and epidermis; however, the number of transcriptional variants varied (Table 2). Nonetheless, the best-matched forms of ALAD (*e*-value = 3.67 × 10^−180^), UROD (*e*-value ≈ 0), and FECH (*e*-value = 4.79 × 10^−165^), i.e., the closest to the canonical forms of the enzymes, were found to be similar between the two organs with respect to sequence and expression, and were used to reconstruct phylogenies amongst metazoans with available data (Figure 8).

The phylogenetic analyses of the main heme biosynthesis proteins demonstrated similarities between sequences from different phyla. The ALAD phylogenetic tree yielded a clear taxonomical organization (Figure 8a). The canonical form from *Eulalia* shared 59% homology with the leech *Helobdella robusta* (another member of the phylum Annelida), and both showed strong homology with the Polychatea *Capitella teleta*. In turn, UROD and FECH sequences (Figure 8b,c respectively) revealed less evident homologies between taxonomically related sequences, which may also have resulted from overall poorer annotation. *Eulalia*’s canonical variants for UROD and FECH revealed high similarity with sequences from organisms not belonging to Annelida, namely *Lingula unguis* from phylum Brachiopoda (UROD) and the sponge *Halichondria panicea* (FECH).

## 3. Discussion

*Eulalia* is known for its bright green coloration, which results from the complex pattern of greenish and yellowish tetrapyrrolic pigments. The pigments are characterized by strong absorption in the UV (280 nm) zone, and in the violet (400 nm) and red (700 nm) regions for yellow and green pigments, respectively. The strong absorption of UV light suggests that these pigments have a role in protecting the worm against UV-induced damage, in a role similar to that of melanins [24]. In fact, *Eulalia* is a known intertidal active forager that seeks shelter from direct sunlight underneath mussel beds [19]. Recent evidence of photoactivation of pigment extracts, especially the yellow pigments from the proboscis, which bear the clearest hallmarks of a porphyrinoid nature, also indicates roles as biocides and potentially even as part of a light-sensing arrangement [23]. We emphasize the yellowish pigment termed Pr2 (from the proboscis) as target for subsequent research due to its abundance, plus its clearer Soret and Q bands. It must be noted, however, that masking of the Soret and Q bands could be explained by porphyrin aggregation and subsequent spectral changes [25]. Nonetheless, we showed that pigment signatures greatly varied between organs. Indeed, while greenish pigments, which were overall the most chemically diverse, appeared to be ubiquitous, yellowish pigments were mostly present in the proboscis.

The two main yellow pigments identified in the proboscis (Pr2) and epidermis (Ep2) seemed to be the same compound, or in some way chemically related. In turn, the green pigments displayed a wider variety and higher interindividual variability. Since heme is the most well-known porphyrin, the absorbance spectra of *Eulalia*’s pigments were compared to the spectra of the heme byproducts biliverdin and bilirubin, which revealed some similarities [26]. Specifically, the yellow pigments had the same absorbance peak as bilirubin near 435 nm, and the green pigments had similar absorbance peaks to biliverdin around 400 nm and 670 nm. Thus, *Eulalia*’s green and yellow pigments are most likely similar to biliverdin and bilirubin, respectively, even though they are seemingly distinct compounds, an issue that needs yet further investigation. The current findings agree with the original study by Martins et al. [22], who identified the major tetrapyrrolic pigments of *Eulalia* and their storage in granules in specialized pigment cells. A few pigments hitherto identified could not be found in the present work—namely the pigment then named Ep1, a yellow pigment from the epidermis (similar to Pr1 from the proboscis), Int2, a green pigment from the intestine, and Oo1, a green pigment from the oocytes. However, Martins et al. [22] also reported that the presence of these pigments was not consistent. Altogether, these differences show an important degree of natural variability. It must also be noted that according to Rodrigo et al. [27], who studied the anatomy and function of the gut of *Eulalia*, pigments are seemingly metabolized and eliminated by the intestinal epithelium, which could explain the variability of the pigments found in this organ. In fact, quite a similar process has been described in the Polychaeta *H. diversicolor,* where the epidermal granules of biliverdin seem to be removed by coelomic cells to the gut for excretion [18]. If a similar translocation process occurs in *Eulalia*, it might also explain the existence of common pigments between the epidermis and the intestine. In addition, the coelomocytes of *Eulalia* are known to transfer nutrients to the oocytes during vitellogenesis [28], from which pigment transfer might be expected as well in *Eulalia* and other Polychaeta. It must be noted, however, that further investigations on the pigments from oocytes were hindered in the current work by difficulties in harvesting sufficient maturing females for analysis, since sampling was done in the fall–winter period; oocyte maturation and vitellogenesis in *Eulalia* takes place in early spring [28]. In any case, it is clear that *Eulalia* and other Polychaeta possess a much wider range and abundance of novel tetrapyrroles than anticipated (far beyond the limited range of vertebrate bile pigments), leading to promising projections for bioprospecting for novel photosensitizers.

**Figure 9:**
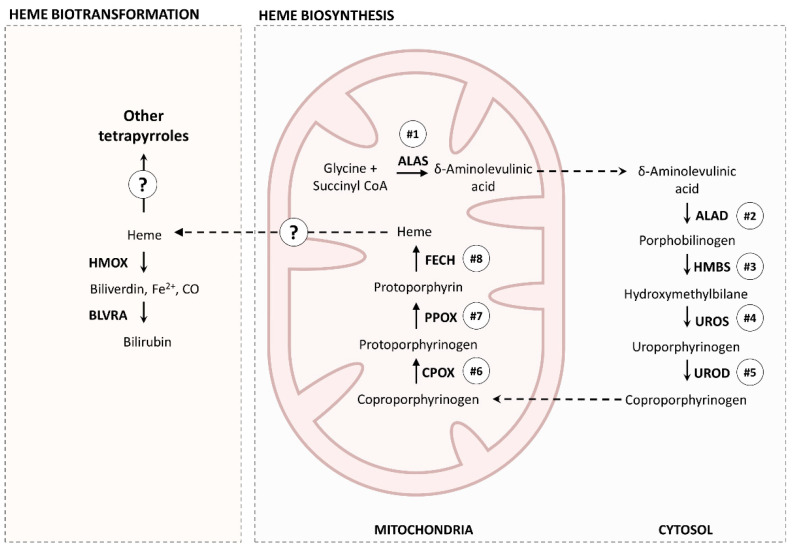
A simplified representation of the essential heme metabolic pathways shared between *Eulalia* and higher-order Eumetazoa, especially vertebrates. ALAS, aminolevulinic acid synthase; ALAD, δ-aminolevulinic acid dehydratase; HMBS, hydroxymethylbilane synthase; UROS, uroporphyrinogen synthase; UROD, uroporphyrinogen decarboxylase; FECH, ferrochelatase; PPOX, protoporphyrinogen oxidase; CPOX, coproporphyrinogen oxidase; HMOX, heme oxygenase; BLVRA, biliverdin reductase.

Even though the metabolism of heme in vertebrates has received much attention in biomedical research, little is known about the metabolism of porphyrins in invertebrates, especially marine annelids, despite their ecological importance, abundance, and evidence for complex pigmentation patterns. Unlike mammals, who possess specific and well differentiated organs and tissues for heme synthesis and degradation, such as bone marrow, spleen, and liver [16,29], *Eulalia* likely relies on pigment cells on the body surface to produce heme or heme-derived metabolites, which explains why the eight canonical enzymes involved in these processes were found in the proboscis and epidermis. Future studies should also address the expression of these genes in the gut epithelium, despite difficulties in extracting high-quality RNA from this organ. These results indicate that the tetrapyrrolic pigments of *Eulalia* are, in fact, heme byproducts, the origins of which may in part relate with the metabolic pathways in vertebrates. These findings align with the chemical characterization of the pigments, and exclude the metabolism of chlorins, the other major class of tetrapyrroles (associated with chlorophylls), as the presence of enzymes for chlorin metabolism was negligible. Still, the differential pigment chemistry between the proboscis and epidermis in *Eulalia* was not circumscribed by the expression of canonical genes, which was similar between the two organs. Instead, the proboscis and epidermis revealed wide differences in gene expression, affecting many metabolic pathways and distinct variants of heme-related gene expression products. The epidermis of *Eulalia* is a relatively extensive and exposed tissue in comparison to the proboscis, which has a more specialized role for predation and sensing [21]; this could explain why more genes were found to be highly expressed in this organ, resulting in a more complex protein network. The enzymes ALAD, UROD, and FECH are involved in the metabolism of heme, since they are involved in the key sequential stages of heme formation. The current findings, therefore, confirm the idea that *Eulalia* possesses the fundamental enzymes involved in the known metabolic pathways of heme (Figure 9). Nonetheless, the existence of complex pigmentation patterns and the absence of pigments clearly identifiable as bilirubin and biliverdin, plus the wide range of heme-related enzymes and their variants that were differentially expressed in the proboscis and the epidermis, indicates that the metabolism of porphyrinoids in these organisms is more intricate than that in vertebrates. On the other hand, only canonical ALAD was clearly clustered within the Annelida (see Figure 8), which is indicative that heme metabolism in *Eulalia* may be a particular case even within this taxon, yielding the synthesis of a particular range of heme-derived metabolites and their intermediate byproducts, which helps to explain the unusual coloration of this species.

The main source of heme for bile pigment synthesis in most vertebrates and some invertebrates, like *H. diversicolor*, derives from the catabolism of hemoglobin [16,18]. However, in *Eulalia*, few blood vessels were observed [21]. This evidence, combined with the outcomes of this study, suggests that *Eulalia* produces heme for hemoglobin on a smaller scale in comparison with the quantity of heme-like products involved in green and yellow pigment production. The enzyme heme oxygenase 2 (HMOX2) was found to be highly expressed in both the proboscis and epidermis, and this observation suggests that these heme-like products are then converted to biliverdin-like metabolites or similar compounds. Biliverdin is usually then rapidly converted to bilirubin by biliverdin reductase (BLVRA), the expression of which was basal and similar between the two organs (data not shown), which may explain the untraceable amounts of bilirubin in the worm and shows that this pathway is likely not a major player in *Eulalia*’s pigment diversity.

The protein networks of the proboscis and epidermis also highlighted differences in various processes within heme metabolism, like pathway regulation and transportation of intermediates. For example, the enzyme ATP-dependent Clp protease ATP-binding subunit clpX-like, mitochondrial (CLPX) was found to be highly expressed in both organs. This enzyme has an important role in the activation of δ-aminolevulinic acid synthase (ALAS) [30], which is the first enzyme in the heme biosynthetic pathway. Additionally, genes coding for proteins involved in porphyrin transportation, namely the broad substrate specificity ATP-binding cassette transporter (ABCG2) and translocator protein (TSPO) were also found to have high levels of expression in both organs. These enzymes have central roles in regulation of the accumulation of the last intermediate of heme biosynthesis, protoporphyrin IX, which is an endogenous photosensitizer [31,32]. Thus, these two enzymes prevent the production of reactive oxygen species (ROS) and subsequent metabolic deregulation. Other enzymes with protective roles against oxidative stress were also found to be highly expressed in both organs, such as glutathione S-transferase A (GSTA1) [33]. Lastly, several cytochromes of the P450 family were significantly represented in the proboscis and epidermis networks, which makes sense as heme is an essential prosthetic group of all P450s [34], and thus benefits from heme recycling and biosynthesis. Interestingly, the most significant and highly expressed enzymes of this family were identified as CYP1A1 (both organs) and CYP1A2 (epidermis), both of which are potentially involved in bilirubin degradation [35]. Altogether, the protein network analysis (Figure 7) indicated the existence of different metabolic pathways between the two organs, expanding the canonical pathways for heme metabolism and catabolism. Even though these mechanisms and their relationships to specific pigments need enlightenment, these differences are seemingly responsible for increased de novo biosynthesis of bilirubin-like photosensitive tetrapyrroles in the proboscis.

## 4. Materials and Methods

### 4.1. Animal Collection

Adult *Eulalia* (pertaining to the *E. viridis*/*E. clavigera* complex) were collected by hand from a rocky intertidal area on Avencas beach, Western Portugal (38°41′17.1″ N 9°21′36.5″ W) between fall and winter of 2019–2020. Animals ranged between 60 and 110 mm in total length and weighed ≈ 280 mg. Individuals were maintained in a mesocosm environment recreating their natural habitat, as described elsewhere [27]. In brief: the mesocosm environment consisted of an aquarium with 7 L of artificial saltwater, protected from direct light and equipped with continuous aeration and recirculation. To provide both shelter and feeding, the aquarium was fitted with natural rocks and clumps of mussels collected from Costa da Caparica beach, a clean area in Western Portugal (38°38′28.0″ N 9°14′18.9″ W). Salinity, temperature, and photoperiod were restricted to 30, 18 °C, and a 10: 14 h light: dark photoperiod, respectively.

### 4.2. Pigment Extraction

Several worms were euthanized by osmotic shock through immersion in ultrapure water. Specimens were immediately microdissected under an optical stereoscope (Leica Microsystems, Wetzlar, Germany) and the target organs were separated, namely the proboscis (Pr), epidermis (Ep), intestine (Int), and, when available, oocytes (Oo) from maturing females. The nomenclature “Epidermis” was used for the worm’s body wall, since the pigments are described to be contained in specialized epidermal cells. The extraction of tetrapyrrolic pigments from biological tissue was done according to the protocol described by Woods and Simmonds [36], with modifications by Martins et al. [22]. In brief: the samples were homogenized in one volume of a mixture of 1 N hydrochloric acid and acetonitrile (1:1) as the extraction solvent (chemicals from Chem-Lab, Zedelgem, Belgium; Carlo Erba Reagents, Barcelona, Spain; respectively). After homogenization, the samples were centrifuged for 5 min at 10,000× *g* at 4 °C to collect the supernatant (fraction containing the pigments). The extraction solvent was then added to the pellet, which was resuspended and homogenized, followed by centrifugation and supernatant collection. The process was repeated until a clear supernatant was obtained, which indicated that no more pigments were available in the sample. The supernatants of each organ were pooled and stored at −20 °C until further analysis. Pigment extraction was performed with minimum exposure to light and air to protect tetrapyrroles from photo-oxidation.

### 4.3. Pigment Characterization

The organic phases of the crude pigment extracts from the proboscis, epidermis, intestine, and oocytes were filtered with a GHP filter (Merck, Darmstadt, Germany) before analysis. Analytical high-performance liquid chromatography (HPLC) was performed to determine the most representative tetrapyrrolic pigments of each target organ. Analyses were conducted with optimizations proposed by Martins et al. [22], based on the separation and quantitation of porphyrins described by Woods and Simmonds [36]. Pigment chromatography was carried out on a Merck-Hitachi L-4500 instrument (Merck, Poole, UK) equipped with a diode array detector (DAD) at a scan range 200–800 nm.

Analytical HPLC was performed with pooled extracts of each target organ from 10 individuals, using a reversed-phase analytical column (RP-HPLC, Phenomenex Onyx Monolithic C18 column, 100 × 4.6 mm i.d., 13 nm, and 2 μm). The injection volume was 20 µL of sample per extract. Optimal peak resolution was obtained with sodium phosphate buffer 10 mM, pH 3.5 (solvent A) and pure methanol (MeOH) (solvent B) at a flow rate of 2 mL/min, using a 10 min linear gradient from 45% to 95% of solvent B and 55% to 5% of solvent A, followed by elution at 95% of solvent B for 2 min. The total run time excluding equilibration was 12 min, and the temperature was maintained at 20 °C and the pressure ≈ 66 bar.

### 4.4. Transcriptome Assembly and Annotation

The raw transcriptome was retrieved from Gene Expression Omnibus (GEO), accession number GSE143954 [37], whereupon extraction and RNA deep-sequencing was performed from the proboscis and epidermis to assemble *Eulalia*’s transcriptome using Trinity v2.8.4 [38]. In brief, as described by Rodrigo et al. [37], total RNA was extracted from the proboscis and epidermis of three individuals. One sample from the proboscis and another from the epidermis were sequenced with high sequencing depth for transcriptome assembly (100 M paired-end reads), and the remaining samples from the two biological replicates were sequenced for normal coverage (20 M paired-end reads). Quantification was done using Kallisto v0.44.0 [39]. To identify the genes expressed in the proboscis and epidermis that coded for proteins associated with the metabolism of porphyrinoids, translated transcripts were annotated based on sequence homology against UNIPROT protein databases with BLASTP [40], having set a maximum *e*-value of 10^−5^. Each database consisted of sequences of proteins associated with the search terms: “chlorins”, “porphyrin Eumetazoa”, “heme biosynthesis”, and “heme degradation”, generating four database subsets.

### 4.5. Gene Expression Profiling

Statistical analysis was done using R 4.0 [41], through packages edgeR and limma. RNA-seq data normalization was performed by creating a DGEList object with the abundance results from the proboscis and epidermis. A linear model was fitted to the transformed data, designed to contrast expression in the proboscis relative to the epidermis. Log_2_ fold-change (logFC) was chosen to display the expression differences. Proboscis transcripts differentially expressed relative to the epidermis were selected with FDR (false discovery rate), adjusted *p*-value < 0.05, and logFC > 2 for overexpressed transcripts and logFC < −2 for underexpressed transcripts. Relative expression within each organ was assessed through logTPM (log_2_ transcripts per million). Expression levels based on logTPM provided a normalized measure of transcript abundance relative to the average abundance of all transcripts in the sample. Thus, a threshold of relative expression was set and transcripts with logTPM > 2 were considered to have high levels of expression, and transcripts with logTPM < −2 were considered to have low levels of expression.

### 4.6. Protein Network Analysis and Homology to Other Metazoa

Networks based on protein–protein interactions related to heme metabolism were inferred from the STRING Database v11.0 [42]. The highly expressed genes in the proboscis and epidermis found in the subsets “porphyrin Eumetazoa”, “heme biosynthesis”, and “heme degradation” were selected to infer differences in the enzymatic process of heme metabolism in both organs. To do so, the organism selected to detect the proteins in the subsets was *Homo sapiens*, due to the high level of genomic annotation. Proteins with no known interactions with other proteins and no association with heme metabolism were removed from the networks. The main heme biosynthesis enzymes represented in both the proboscis and epidermis were validated by sequence comparison to other Metazoa organisms with MrBayes [43], using the maximum likelihood method with a LG gamma distribution model (1,000,000 iterations). Sequence alignment (by ClustalW) and testing for best model were conducted in MEGA X [44].

### 4.7. Validation by RT-qPCR

Results from the transcriptomics approach were validated by reverse-transcription quantitative polymerase chain reaction (RT-qPCR) for mRNAs coding for relevant heme biosynthesis proteins. Total RNA was extracted from the proboscis and epidermis of three individuals. The extraction was done on samples stabilized with RNALater using the Rneasy Protect Mini Kit (QIAGEN, Hilden, Germany) for purification of total RNA from animal tissues. Synthesis of cDNA was done by reverse-transcription using the First-Strand cDNA Synthesis Kit (NZYTech, Lisbon, Portugal) according to the manufacturer’s instructions. Primers were designed with Primer Blast against *Eulalia*’s sequences of best-matched target mRNAs and verified with Oligo Analyzer. The RT-qPCR protocol was completed in a Corbett Rotor-Gene 6000 thermal cycler (QIAGEN) with the NZY qPCR Green Master Mix (NZYTech, Lisbon, Portugal), following the manufacturer instructions. The programmed settings were, with 45 cycles per run, denaturation at 94 °C for 45 s, annealing at 55 °C for 35 s, and extension at 72 °C for 30 s. As a calibrator, glyceraldehyde-3-phosphate dehydrogenase (GAPDH) was used, as suggested by Thiel et al. [45]. Relative expression was determined using the 2^−∆∆Ct^ method [46]. Statistical analysis was conducted with Student’s *t*-test and Mann–Whitney *U* test as parametric and nonparametric procedures, respectively. Normality and homoscedasticity were analyzed using Shapiro and Levene tests, respectively. Statistics were performed with R 4.0 [41].

## 5. Conclusions

To summarize, the present findings indicate that the proboscis and epidermis of *Eulalia* share conserved heme metabolic pathways. These include proteins involved in heme biosynthesis, degradation, and transformation, as well as proteins for pathway regulation and transportation described for higher-order organisms, particularly vertebrates. Even though this outcome highlights the fact that porphyrinoid metabolism is well conserved among the animal kingdom, it does not explain the species’ wide variety of endogenous tetrapyrroles and the patterns of pigmentation that are clearly distinct between the proboscis and the epidermis. Instead, the combination of transcriptomics and porphyrinoid metabolomics suggests the existence of complex metabolic pathways that include novel enzyme variants of the canonical enzymes involved in the synthesis, catabolism, and transport of porphyrinoids that differ between organs. Most importantly, the consistency of pigment signatures of the proboscis and epidermis of these organisms reflects their primary coloration rather than a stochastic or environment-dependent accumulation of secondary metabolites, as observed for *Hediste*. Altogether, it is clear that *Eulalia* is well equipped to metabolize and recycle heme into a far wider range of tetrapyrrolic pigments, conferring upon the species an adaptive leverage towards its specific environment, which includes novel bilirubin-like photosensitizers that play an important role in sensing and protection against biotic and abiotic agents. Although much work is needed to isolate and characterize individual pigments, their intermediates, and their relationships with the functions of specific enzyme proteoforms, the discovery of essential enzymes involved in pigment synthesis and biotransformation breaks new ground toward mapping metabolic pathways in order to develop efficient and cost-effective synthetic processes in vitro and ex vivo in the future.

## Figures and Tables

**Table 1:** Summary of main pigments in *Eulalia* by organ. Each pigment was selected according to pigment absorption maxima. Retention time (min) corresponds to the timeframe within which the pigment was eluted from the HPLC column. Pigment color was determined by visual inspection and absorption at 400 nm (for yellow pigments) and 700 nm (for green pigments). Similar pigments are identified as a, b, or c (see Figure 2 as well).

Organ	Pigment ID	Color	Retention Time (min)	
Proboscis	Pr1	Yellow	1.04–1.63	
	Pr2	Yellow	3.69–4.34	a
Epidermis	Ep2	Yellow	3.85–4.25	a
	Ep3	Green	6.36–6.51	
	Ep4	Green	6.61–6.76	c
Intestine	Int1	Green	5.41–5.56	b
	Int3	Green	6.63–6.72	c
	Int4	Green	4.01–4.32	
Oocytes	Oo2	Green	4.70–5.01	
	Oo3	Green	5.37–5.53	b
	Oo4	Green	5.64–5.74	
	Oo5	Green	6.56–6.70	c

**Table 2:** Comparative numbers of highly abundant transcriptional variants (logTPM > 2) of heme-metabolism-related enzymes in the proboscis and epidermis of *Eulalia* after homology matching against the three Uniprot subsets (“porphyrin Eumetazoa”, “heme biosynthesis”, and “heme degradation”).

Subset	Enzymes	Proboscis	Epidermis	Proboscis and Epidermis
Porphyrin Eumetazoa	ALAD	2	2	16
	UROD	2	2	3
	FECH	8	5	8
Heme biosynthesis	ALAD	0	0	1
	UROD	2	4	4
	FECH	8	6	15
	HMOX2	0	0	1
Heme degradation	HMOX2	0	0	1

ALAD, δ-aminolevulinic acid dehydratase; UROD, uroporphyrinogen decarboxylase; FECH, ferrochelatase; HMOX2, heme oxygenase 2.

## Data Availability

Full transcriptome data can be freely accessed at Gene Expression Omnibus, accession GSE143954 (https://www.ncbi.nlm.nih.gov/geo/query/acc.cgi?acc=GSE143954).

## Sample Availability

Samples of the compounds are not available from the authors.
